# RT-NET: real-time reconstruction of neural activity using high-density electroencephalography

**DOI:** 10.1007/s12021-020-09479-3

**Published:** 2020-07-28

**Authors:** Roberto Guarnieri, Mingqi Zhao, Gaia Amaranta Taberna, Marco Ganzetti, Stephan P. Swinnen, Dante Mantini

**Affiliations:** 1 Research Center for Motor Control and Neuroplasticity, KU Leuven, Tervuursevest 101, 3001 Leuven, Belgium; 2 Roche Pharmaceutical Research and Early Development, Roche Innovation Center, 4051 Basel, Switzerland; 3 Leuven Brain Institute, KU Leuven, 3000 Leuven, Belgium; 4 Brain Imaging and Neural Dynamics Research Group, IRCCS San Camillo Hospital, 30126 Venice, Italy

**Keywords:** Electroencephalography, Neural activity, Online processing, Head model, Source localization

## Abstract

High-density electroencephalography (hdEEG) has been successfully used for large-scale investigations of neural activity in the healthy and diseased human brain. Because of their high computational demand, analyses of source-projected hdEEG data are typically performed offline. Here, we present a real-time noninvasive electrophysiology toolbox, RT-NET, which has been specifically developed for online reconstruction of neural activity using hdEEG. RT-NET relies on the Lab Streaming Layer for acquiring raw data from a large number of EEG amplifiers and for streaming the processed data to external applications. RT-NET estimates a spatial filter for artifact removal and source activity reconstruction using a calibration dataset. This spatial filter is then applied to the hdEEG data as they are acquired, thereby ensuring low latencies and computation times. Overall, our analyses show that RT-NET can estimate real-time neural activity with performance comparable to offline analysis methods. It may therefore enable the development of novel brain–computer interface applications such as source-based neurofeedback.

## Introduction

Functional magnetic resonance imaging (fMRI) is currently the primary research tool for investigating human brain function (Fox and Raichle 2007; Ganzetti and Mantini 2013). However, fMRI only provides an indirect measure of neural activity mediated by a slow hemodynamic response. Electroencephalography (EEG) is a brain imaging technique alternative to fMRI. EEG measures changes in electric potentials over the scalp, which are generated by neuronal currents flowing through the head (Speckmann et al. 2012). Notably, source activity reconstruction using EEG requires realistic biophysical models that incorporate the exact positions of EEG electrodes as well as the anatomical properties of an individual’s head (Brett et al. 2002). Estimation of brain sources from EEG recordings (Ganzetti and Mantini 2013; Pfurtscheller and Lopes Da Silva 1999) is typically referred to as the *inverse problem*. On the other hand, the assessment of EEG recordings from brain sources is referred to as the *forward problem *(Hallez et al. 2007).

To date, several software solutions have been made available to the neuroscientific community for offline analysis of EEG recordings, including EEGLab (Delorme and Makeig 2004), Fieldtrip (Oostenveld et al. 2011), Brainstorm (Tadel et al. 2011), SPM (Litvak et al. 2011) and MNE (Gramfort et al. 2014). Recently, our research group proposed an offline analysis workflow specifically suited for high-density (hdEEG) data, which integrates several tools from existing software with original solutions for data preprocessing, realistic head model generation and source localization. So far, our analysis workflow for hdEEG has been used to reconstruct large-scale brain networks (Liu et al. 2017, 2018) and to examine functional connectivity between network nodes (Samogin et al. 2019). Such an application does not require online data processing, which is instead needed for brain–computer interface (BCI) studies. Real-time reconstructions of source-space EEG activity could enhance the effectiveness of BCI applications, such as neurofeedback (Boe et al. 2014; van Lutterveld et al. 2017). MNE Scan (https://www.mne-cpp.org/index.php/category/development/mne-scan) and NeuroPype (https://www.neuropype.io) have been recently introduced as new software packages for online analysis of EEG data. They offer several tools for real-time EEG data processing and feature extraction, and also incorporate source localization tools. They are not optimized for hdEEG systems as they rely on a template head model that does not consider electrode positions collected during the same experimental session (Van Hoey et al. 2000).

To address the limitation described above, we introduce a novel software package for Real-Time Noninvasive Electrophysiology (RT-NET), which is distributed under a GNU General Public License (GPL). RT-NET permits online neural activity reconstruction from hdEEG recordings. The user can access the different analysis steps through a graphical user interface (GUI). Unlike MNE Scan and NeuroPype, RT-NET permits the generation and use of a realistic head model based on electrode positions collected just before EEG recordings, leading to an enhanced precision in neural activity reconstruction. To ensure very short processing times, it relies on an adaptive spatial filter for artifact attenuation as well as for source localization. In the present study, we assessed the effectiveness and validity of RT-NET on hdEEG data collected during hand movements. Specifically, we compared the neural activity reconstructed online with that estimated by an offline analysis workflow.

## Methods

**Fig. 1.**
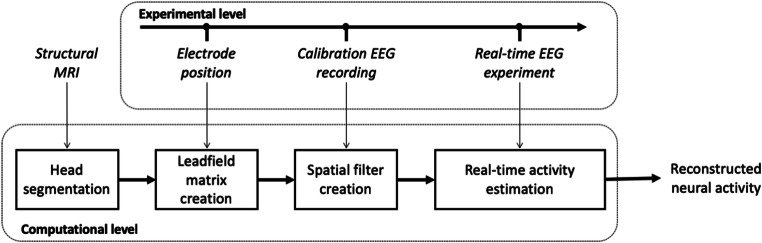
RT-NET modules and their execution during a typical hdEEG experimental session. Prior to the hdEEG session, the anatomical MR image is segmented into three tissue classes. During hdEEG, the participant wears the hdEEG cap and the electrode positions over the scalp are recorded. After the co-registration of the complete set of electrode positions over the MR image, the leadfield matrix is generated. A calibration recording is acquired in the participant. This recording is used to estimate an *artifact attenuation filter*, *F*_0_, which reduces noise and non-neuronal signals, and a *source localization filter*, *K*, for reconstructing neural activity in the source-space. Finally, during the real-time EEG experiment, the spatial filter is applied to the hdEEG data, generating the reconstruction of active brain sources in an online modality.

RT-NET was written using the MATLAB (The Mathworks, Natick, MA, US) programming environment. Therefore, existing libraries and functions for EEG data analysis such as EEGLab, Fieldtrip, Brainstorm, SPM and Lab Streaming Layer (LSL) can be easily integrated. The source code and the software manual can be downloaded using the following links: https://www.nitrc.org/projects/rtnet or https://github.com/robertoguarnieri/rtnet. Being the source code available, software customization or extension is possible. The documentation specifies the software requirements and guides the user through the whole processing pipeline. RT-NET has been specifically developed for optimal integration with the stages of a classical hdEEG experiment (Liu et al. 2017; Michel and Brunet 2019), such as the collection of a magnetic resonance (MR) image, of electrode positions, as well as of hdEEG data (Fig. 1).

### Toolbox Description

**Fig. 2:**
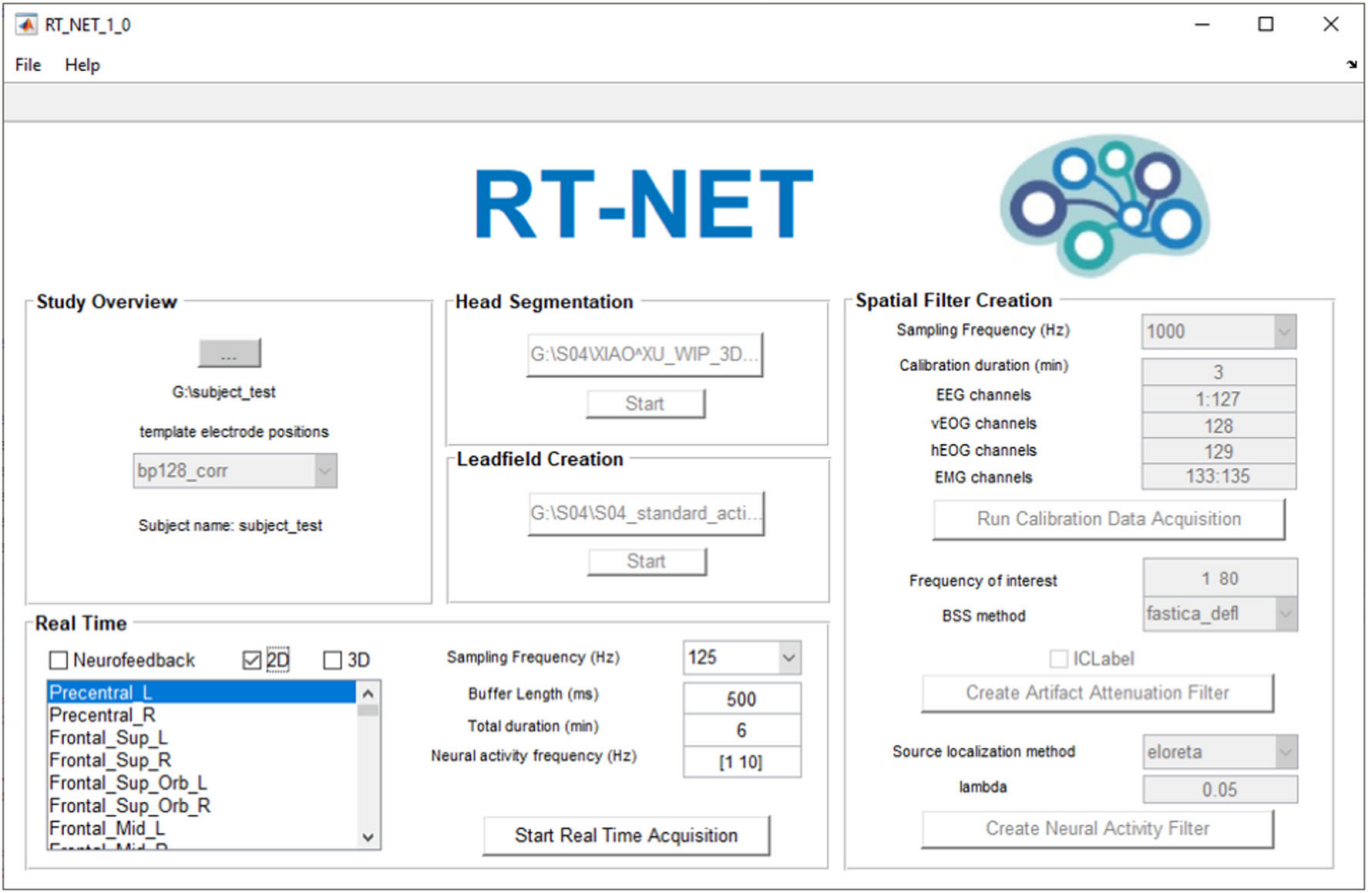
RT-NET graphical user interface. *Study Overview* is the first panel of the toolbox, in which it is possible to load the output folder of the study and visualize the information. After that, the user can load the structural image of the subject’s head, previously acquired, and start the *Head Segmentation*. The *Leadfield creation* step starts when the file containing the electrode positions over the subject’s scalp are loaded. For the *Spatial Filter Creation* step, the user needs to acquire a hdEEG calibration recording. A filter for real-time neural reconstruction is initialized using this recording. Finally, the user can enable and customize the online reconstruction of neural activity by using the *Real-Time* panel

As already mentioned, the GUI of RT-NET gives access to all the functions required for online brain activity reconstruction. Therefore, there is no need for the user to have programming experience. The GUI offers indeed a simplified, structured and user-friendly tool (Fig. 2).

The GUI is organized in five different modules: *Study overview*, *Head segmentation*, *Leadfield matrix creation*, *Spatial filter creation* and *Real-time activity estimation*. Each time an analysis step is completed through a module of RT-NET, a new panel is activated. First, in the *Study Overview* window*,* the user, after the initialization of a new project, can choose the output folder and the type of EEG system that will be used. Before the EEG session, an anatomical image of the participant’s head needs to be acquired using MR imaging, or alternatively, a template included in the software’s directory can be used. Through the *Head segmentation* module, the structural MR image is segmented into different tissue classes. Next, the EEG cap is positioned over the participant’s scalp and the electrode positions are recorded. After the co-registration of the complete set of electrode positions over the MR image and the generation of a realistic volume conductor model, the *Leadfield matrix creation* tool calculates the linear relationships between neural currents in the brain (sources) and electric potentials measured at the electrode level*.* The *Spatial filter creation* module can be initialized through the acquisition of an EEG calibration recording. By using this recording, a spatial filter is produced, which is capable of attenuating artifactual signals in the hdEEG data and estimating neural activity in the brain. With the *Real-time activity estimation* module, the spatial filter is applied to the EEG data, supporting the online reconstruction of brain sources. A detailed description of RT-NET modules is provided here below.

#### Initialization and Study Overview

When the GUI is launched, the user is required to initialize a new project through the *Study Overview* module. This permits the user to choose the output folder for saving the data and the EEG system that will be used for the experiment. The EEG systems that are recognized by RT-NET are those compatible with LSL (https://github.com/sccn/labstreaminglayer), an open-source software solution for communicating with external devices in real-time and with millisecond accuracy. It should be noted that, although LSL is platform-independent, it is more easily accessible in its Windows implementation. This may also result in an easier use of RT-NET with the Windows operating system. After defining the input required by the *Study Overview* module, it is possible to start the *Head Segmentation* module.

#### Head Segmentation

The second module of RT-NET, *Head Segmentation*, is designed to automatically perform the segmentation of the MR image of the subject’s head. The estimation of EEG signals from the brain sources (also known as the *forward solution*) requires a realistic head-volume conductor model to be generated from an individual’s MR image, and the correct electrode locations to be defined with respect to the conductor model. Therefore, the first processing stage of our workflow involves the segmentation of the MR image into three different tissue classes: brain, skull and skin (Gramfort et al. 2010). This is done using the unified segmentation algorithm implemented in SPM12 (Ashburner and Friston 2005) (https://www.fil.ion.ucl.ac.uk/spm/software/spm12/). The choice of segmenting the brain in three tissue classes is due to the need of balancing realistic modelling and computational efficiency (Fuchs et al. 2002).

The tissue probability maps produced by SPM12 are then binarized using a winner-takes-all approach (Ashburner and Friston 2005). Finally, the three tissue compartments in the volume space are processed with Fieldtrip (http://www.fieldtriptoolbox.org) to build hexahedral meshes.

#### Leadfield Matrix Creation

Once the head segmentation is performed, the *Leadfield matrix creation* window is activated. First, the electrode positions file, previously acquired, needs to be loaded and then the computations can start. This preprocessing module allows the generation of the leadfield matrix, *L*, containing the scalp potentials putatively measured for each possible configuration of neural source position and orientation. Specifically, the scalp potential *θ*, can be expressed as follows (Pascual-Marqui et al. 2011):

1 $$ \theta (t)=L\cdotp J(t) $$

where, if *n*_*E*_ is the number of electrodes and *n*_*V*_ the number of voxels, then the leadfield matrix, *L*, has dimension [*n*_*E*_ · 3*n*_*V*_]; the current density *J*(*t*) has dimension [3*n*_*V*_ · 1]; and θ(*t*) has dimension [*n*_*E*_ · 1].

The first step for leadfield matrix creation is the co-registration of the electrode positions in the same space, defined by the MR image. To this end, a rigid-body transformation matching the landmarks in the electrode space to the corresponding ones in the MR space is computed and applied to the electrode positions. These are then aligned to the head surface extracted from the individual MR image using the iterative closest point algorithm (Besl and McKay 1992) implemented in SPM12. Finally, each electrode is orthogonally projected onto the head surface.

The second step is the creation of the volume conductor model. The meshes derived from MR images for the brain, skull and skin compartments are used, along with the conductivity values defined for each of them. These are set by default to 0.33, 0.01 and 0.43, respectively, in line with the relevant literature (Haueisen et al. 1997; Holdefer et al. 2006), but can also be modified by the user if needed.

The electrode positions and the volume conduction model are combined to create the leadfield matrix by numerical simulations, which are conducted using the symmetric boundary element method (sBEM) implemented in OpenMEEG (Gramfort et al. 2010, 2011). The leadfield matrix, initially computed for each mesh element spanning the gray matter, is then resampled in the volume space, defined as a regular volumetric grid with 6-mm resolution.

#### Spatial Filter Creation

The *Spatial filter cre*ation module permits the generation, from a hdEEG calibration recording, of a spatial filter that will subsequently be used for online artifact reduction and source localization. First, the user needs to choose the length of the calibration recording (in minutes), the sampling frequency (in Hz), the frequency band of interest (in Hz), the EEG channels, the data decomposition technique, the auxiliary electrical channels (i.e. electrooculogram or electromyogram) to be used for artifact detection, the source localization method and its parameters. Next, the calibration recording can be acquired by pressing the corresponding button.

First of all, we detect channels with low signal quality and label them as ‘bad channels’. To this end, we use an automated procedure that combines information from two different parameters. The first parameter is the minimum Pearson correlation of the signal in the frequency band of interest selected by the user, against all the signals from the other channels. The second parameter is the noise variance in the band 200-250 Hz, where the contribution of the EEG signal can be considered negligible. We define the list of bad channels *b*, including those channels for which at least one of the two channel-specific parameters are outliers as compared to the total distribution of values. To ensure robustness of the detection, the threshold to define an outlier is set to *m* + 4*s*, where *m* is the average value and *s* is the standard deviation. Subsequently, the list of neighboring channels is defined using the FieldTrip toolbox (http://www.fieldtriptoolbox.org). A *channel adjacency matrix D* with dimension [*n*_*E*_ · *n*_*E*_], is created, with each element *d*_*ij*_ equal to 1 if channels *i* and *j* are adjacent (and not labelled as ‘bad channels’), and equal to 0 otherwise. A *bad-channel correction matrix C,* with dimension [*n*_*E*_ · *n*_*E*_], is then created:

2 $$ {c}_{ij}=\left\{\begin{array}{c}{d}_{ij}/\sum \limits_j{d}_{ij}\kern0.75em if\ i\in b\\ {}1\kern0.75em if i\notin b\kern0.5em and\ i=j\\ {}0\kern0.75em if i\notin b\kern0.5em and\ i\ne j\end{array}\right.. $$

Next, we generate a data re-referencing matrix *R* to perform average re-referencing (Liu et al. 2015). The matrix *R*, with dimension [*n*_*E*_ · *n*_*E*_], is defined as follows:

3 $$ {r}_{ij}=\left\{\begin{array}{c}\left({n}_E-1\right)/{n}_E\kern0.75em if\ i=j\\ {}-\frac{1}{n_E}\kern0.5em if\ i\ne j\end{array}\right.. $$

We apply the spatial filters described by matrices *C* and *R* to the raw EEG data *X*(*t*), so to obtain a new EEG dataset *X*_*q*_(*t*), in which all signals are in average reference and those from bad channels are repaired:

4 $$ {X}_q(t)=C\cdotp R\cdotp X(t). $$

Using EEGLab (https://sccn.ucsd.edu/eeglab), we band-pass filter the resulting EEG data in the frequency range selected by the user, so to obtain the new EEG dataset ${\overset{\sim }{X}}_q(t)$. Then, we apply independent component analysis (ICA) (Mantini et al. 2008) using a fast fixed-point ICA (FastICA) algorithm (http://research.ics.aalto.fi/ica/fastica) in deflation approach and with hyperbolic tangent as contrast function (Hyvarinen 1999). Other ICA algorithms are implemented in RT-NET, and can be alternatively used: FastICA in symmetric approach, Infomax (Lee et al. 1999) and JADE (Cardoso 1999). The ICA model can be described as:

5 $$ {\overset{\sim }{X}}_q(t)=A\cdotp S(t) $$

where ${\overset{\sim }{X}}_q=\left[{X}_1(t),\dots, {X}_{n_E}(t)\right]$ is the matrix of *n*_*E*_ observed signals; $S(t)=\left[{S}_1(t),\dots, {S}_{n_S}(t)\right]$ is the matrix of *n*_*S*_ underlying signals, or independent components (ICs); *A*, with dimension [*n*_*E*_ · *n*_*S*_], denotes the mixing matrix (Stone 2004). The ICs can be retrieved by determining the unmixing matrix *W,* with dimension [*n*_*S*_ · *n*_*E*_], such that:

6 $$ S(t)=W\cdotp {\overset{\sim }{X}}_q(t). $$

After that FastICA has been run on the EEG calibration dataset ${\overset{\sim }{X}}_q(t)$, the ICs associated with the artifacts (or artifactual ICs) are automatically identified. This can be done either using ICLabel (https://sccn.ucsd.edu/wiki/ICLabel) (Pion-Tonachini et al. 2019), or the IC artifact detection solution implemented in Liu et al. (2017). The latter, which is the default solution in RT-NET, relies on the following parameters: 1) correlation between the power of the IC with vertical electrooculogram (vEOG), horizontal electrooculogram (hEOG) and electromyogram (EMG); 2) the coefficient of determination obtained by fitting the IC power spectrum with a 1/*f* function; 3) the kurtosis of the IC. An IC is classified as artifactual if at least one of the above parameters is above its specific threshold, set in accordance with previous studies (De Pasquale et al. 2010; Liu et al. 2017; Mantini et al. 2009). The unmixing matrix *W*_*A*_ for the artifactual components *S*_*A*_(*t*) is obtained by selecting the corresponding rows of the matrix *W*, such that:

7 $$ {S}_A(t)={W}_A\cdotp X(t). $$

An *artifact attenuation filter F*_0_, with dimension [*n*_*E*_ · *n*_*E*_], is initialized as:

8 $$ {F}_0=I-X\cdotp {S}_A^T\cdotp {\left({S}_A\cdotp {S}_A^T\right)}^{-1}\cdotp {W}_A $$

where *I* is an identity matrix with dimension [*n*_*E*_ · *n*_*E*_].

The artifact-free calibration dataset ${\overset{\sim }{X}}_p(t)$ is generated by applying the initial artifact attenuation filter *F*_0_ to ${\overset{\sim }{X}}_q(t)$, as follows:

9 $$ {\overset{\sim }{X}}_p(t)={F}_0\cdotp {\overset{\sim }{X}}_q(t). $$

It should be noted that the *artifact attenuation filter F*(*t*) is dynamically defined during the acquisition of real hdEEG data, following the approach described in Guarnieri et al. (2018). This approach is explained in detail in the next section, dedicated to online data analysis.

Using the artifact-free calibration dataset ${\overset{\sim }{X}}_p(t)$, a *source localization filter K*, with dimension [3*n*_*V*_ · *n*_*E*_], is also created. This specific filter depends on the selected source localization algorithm. RT-NET integrates the exact low-resolution brain electromagnetic tomography (eLORETA) algorithm (Pascual-Marqui et al. 2011) as default solution. In this case, the source localization filter *K* is calculated using the following formula:

10 $$ K={G}^{-1}\cdotp {L}^T\cdotp {\left(L\cdotp {G}^{-1}\cdotp {L}^T+\alpha H\right)}^{+} $$

where *L* is the leadfield matrix, *G* is a symmetric positive definite weight matrix with dimension [3*n*_*V*_ · 3*n*_*V*_], *H* is the noise covariance matrix estimated from ${\overset{\sim }{X}}_p(t)$, *α* > 0 is the Tikhonov regularization parameter and ^*+*^ denotes the Moore–Penrose pseudoinverse. The regularization parameter *α* is set by default to 0.05 and can be changed by the user if needed. Other source localization algorithms implemented in RT-NET are the standardized low resolution brain electromagnetic tomography (sLORETA) algorithm (Pascual-Marqui 2002), the minimum norm estimates (MNE) (Hämäläinen and Ilmoniemi 1994), its weighted version wMNE (Lin et al. 2006) and the linearly constrained minimum variance beamformer (LCMV) (Van Veen et al. 1997). All the source localization methods above are implemented in volumetric space. In particular, eLORETA, LORETA, MNE and LCMV are those integrated in FieldTrip (http://www.fieldtriptoolbox.org), whereas sLORETA and wMNE are those in Brainstorm (https://neuroimage.usc.edu/brainstorm).

#### Real-Time Activity Estimation

The *Real-time activity estimation* module allows the reconstruction of ongoing neural activity for all the voxels in the gray matter or, alternatively for selected regions of interest (ROIs), by using the spatial filters created using the calibration recording. The parameters that need to be defined before real-time activity estimation are: buffer length (in ms), total duration of the experiment (in minutes), sampling frequency and the frequency band of interest for neural activity estimation (both in Hz). Furthermore, it is necessary either to select the ROIs for which neural activity needs to be extracted, or to enable reconstruction in each voxel of the gray matter for real-time mapping of neural activity.

Within the real-time activity estimation module, EEG data are stored in a buffer with *n*_*T*_ samples, determined based on the sampling frequency and the buffer length set by the user. The EEG data in the buffer *X*(*τ*) is filtered in the frequency band of interest, thereby obtaining $\overset{\sim }{X}\left(\tau \right)$ . Next, the *bad-channel correction matrix C* and the *re-referencing matrix R* are applied*:*

11 $$ {\overset{\sim }{X}}_q\left(\tau \right)=C\cdotp R\cdotp \overset{\sim }{X}\left(\tau \right). $$

Starting from the resulting dataset ${\overset{\sim }{X}}_q\left(\tau \right)$, we estimate artifactual signals that are present in the buffer, using the matrix *W*_*A*_ obtained from the calibration dataset:

12 $$ {S}_A\left(\tau \right)={W}_A\cdotp {\overset{\sim }{X}}_q\left(\tau \right). $$

At this point, linear regression analysis is used to estimate the weight matrix *B*_*A*_ associated with the artifactual signals in the buffer. In particular, the following equation is considered to account for the non-stationarity of the artifactual contribution in the EEG signals:

13 $$ {\overset{\sim }{X}}_q\left(\tau \right)={B}_A\ast {S}_A\left(\tau \right)+\varepsilon \left(\tau \right) $$

where *ε*(*τ*) is the residual of *X*(*τ*) that cannot be explained by a linear combination of *S*_*A*_(*τ*). Using the method proposed in Guarnieri et al. (2018), an adaptive spatial filter *F*(*τ*) is built to dynamically obtain artifact-free signals ${\overset{\sim }{X}}_p\left(\tau \right)$, such that:

14 $$ {\overset{\sim }{X}}_{\mathrm{p}}\left(\tau \right)=F\left(\tau \right)\cdotp {\overset{\sim }{X}}_q\left(\tau \right) $$

where *F*(*τ*) is defined as follows:

15 $$ F\left(\tau \right)=I-X\left(\tau \right)\ast {S}_A^T\left(\tau \right)\ast {\left({S}_A\left(\tau \right)\ast {S}_A^T\left(\tau \right)\right)}^{-1}\ast {W}_A. $$

Considering that the buffer is dynamically updated at the same frequency as the sampling rate, the latest sample in the artifact-cleaned EEG dataset ${\overset{\sim }{X}}_p\left(\tau \right)$ is continuously extracted to estimate real-time neural activity in the sensor space *Y*(*t*).

When the reconstruction of neural activity from ROIs is selected, the primary voxel indices corresponding to the ROIs are identified and the source localization matrix *K* is downsampled accordingly. In this case, the dimension of matrix *K* becomes [3*n*_*R*_ · *n*_*E*_], where *n*_*R*_ is the number of ROIs, and the neural signals are separately reconstructed for the three directions. The *source localization filter K* is then applied to *Y*(*t*), such that real-time neural activity in the source space *B*(*t*) is also obtained:

16 $$ B(t)=K\cdotp Y(t). $$

By default, the artifact-free signals in the sensor space *Y*(*t*), and in the source space *B*(*t*), are forwarded to LSL for real-time visualization or control of other devices, such as a brain stimulation system for closed-loop applications (Boe et al. 2014; Semprini et al. 2018). These reconstructed neural signals are also saved in the *output folder,* to be analyzed offline.

### Validation of RT-NET

We assessed the performance of the RT-NET toolbox using real hdEEG data. We compared the signals processed with RT-NET against those obtained with our offline analysis workflow (Liu et al. 2017). Specifically, we focused on the modulations of neural activity induced by movements of the right hand (Weiss et al. 2013).

#### Data Collection

Data used in this study were obtained from hdEEG recordings collected in 10 healthy right-handed participants (five men and five women, age range 23–39 years). All participants reported normal or corrected-to-normal vision and had no psychiatric or neurological history. They gave written informed consent to the experimental procedures, which were approved by the Institutional Ethics Committee of KU Leuven.

In a first experimental session, a structural T1-weighted MR image of the participant’s head was collected with a 3 T Philips Achieva MR scanner (Philips Medical Systems, Best, Netherlands) using a magnetization-prepared rapid-acquisition gradient-echo (MP-RAGE) sequence (Mugler and Brookeman 1991). The scanning parameters were TR = 9.6 ms, TE = 4.6 ms, 160 coronal slices, 250 × 250 matrix, and voxel size 0.98 × 0.98 × 1.2 mm^3^. The MR image was used during the EEG experimental session to generate the volume conduction model for source localization.

In a second experimental session, electrode positions were first acquired using the Xensor system (ANT Neuro, Enschede, Netherlands). Subsequently, two hdEEG datasets were collected: the first one, which was used for spatial filter creation using RT-NET, with the participant being at rest for 4 min; the second one with them performing right-hand movements for 6 min. hdEEG signals were sampled at 1 kHz using the 128-channel actiCHamp system (Brain Products GmbH, Gilching, Germany). The electrode at vertex (Cz in the 10/20 international system) was used as the physical reference. In addition, we also recorded horizontal and vertical EOG (hEOG and vEOG) as well as three electromyography (EMG) signals associated with the masseter (right), trapezius (right), splenius capitis (right) and carpi radialis longus (right) muscles. The first three EMG signals were used for artifact removal, whereas the fourth EMG signal was used to detect hand movement onsets. For the resting-state part of the EEG session, participants were asked to fixate on a black cross in the center of a white screen (eyes-open fixation). In the motor-related part of the EEG session, participants were asked to perform right wrist flections/extensions, alternating 6 s of self-paced uninterrupted movements with 6 s of eyes-open fixation.

#### Analysis of RT-NET Performance

A crucial metric to assess the performance of the toolbox is the computational time. This was quantified using a computer with a 2.5-GHz Intel Core i7 processor and 16 GB RAM, running Windows 10. We quantified the time required for the *Head segmentation*, *Leadfield matrix creation* and *Spatial filter creation* modules of RT-NET. We also evaluated the computational delay during online hdEEG acquisition and processing. This analysis was conducted using a buffer length of 500 ms, as in Guarnieri et al. (2018), band-pass frequency between 1 and 50 Hz, and sampling frequency equal to 100 Hz.

The neural signals reconstructed in real-time using the *Real-time reconstruction* module were used to produce spatial maps reflecting event-related synchronization/desynchronization (ERS/ERD) maps across trials. ERD/ERS can be expressed using the following formula:

17 $$ ERD\left(f,t\right)=\frac{P\left(f,t\right)-{P}_b(f)}{P_b(f)}\cdotp 100\% $$

where *P*(*f*, *t*) is the power in a given frequency band and time interval, and *P*_*b*_(*f*) is the average power over time in a baseline period (Pfurtscheller and Lopes Da Silva 1999). ERD maps were calculated for the beta band (13–30 Hz) in the period [0 s, +2 s] with respect to movement onset. The beta band was chosen, as it is typically implicated in motor execution (Pfurtscheller and Lopes Da Silva 1999). The baseline period [−1 s, 0 s] was defined with respect to the same onset. The ERD maps were visualized in real-time using a 3D cortical model with 3500 vertices, which was generated using FieldTrip (Oostenveld et al. 2011). The correlation between ERD maps was calculated offline after the experiment, to quantify the reliability of the results across trials.

After verifying the feasibility of using RT-NET in a real-time hdEEG experiment, we also quantified the accuracy of source localization. To this end, we used an offline analysis as a benchmark. The offline analysis workflow was the same applied to the calibration EEG dataset, and included bad-channel correction, re-referencing, band-pass filtering, ICA-based artifact removal, head modelling using sBEM and source localization using eLORETA (Liu et al. 2017, 2018). The reliability of task-related modulations in neural activity was assessed using the average ERD map across trials, again for the beta band. We also conducted an ERD analysis for selected ROIs, whose MNI coordinates were chosen on the basis of relevant fMRI studies (Debaere et al. 2003, 2004; Gorgolewski et al. 2013; Lv et al. 2013; Rémy et al. 2008; Weiss et al. 2013). The ROIs were the left primary motor cortex (M1; MNI coordinates [−38, −20, 58]), the supplementary motor area (SMA; [0, −4, 56]), the left ventral premotor cortex (VPMC; [−30, −10, 58]), and the left superior temporal gyrus (STG; [−58, −32, 6]). The latter, whose activity is expected to be minimally modulated by motor task performance, was used as the control ROI. For each ROI, the MNI coordinates were converted to individual space. Spherical ROIs with a radius of 6 mm were then created (Marrelec and Fransson 2011). Neural signals from the ROIs were extracted, and first used to assess the presence of residual artifacts in the source-localized data. This was quantified using the absolute temporal correlation between reconstructed neural signals and simultaneously collected EOG and EMG signals. By using temporal correlations, we also compared ERD time-courses obtained using RT-NET and the offline analysis workflow, either in the beta band (13–30 Hz) and in the full band (1–50 Hz). This permitted us to estimate the presence of motor-related activity in the reconstructed neural signals. A Wilcoxon signed rank test was carried out to assess significant differences.

## Results

### Computation Time for RT-NET Analysis

**Fig. 3.**
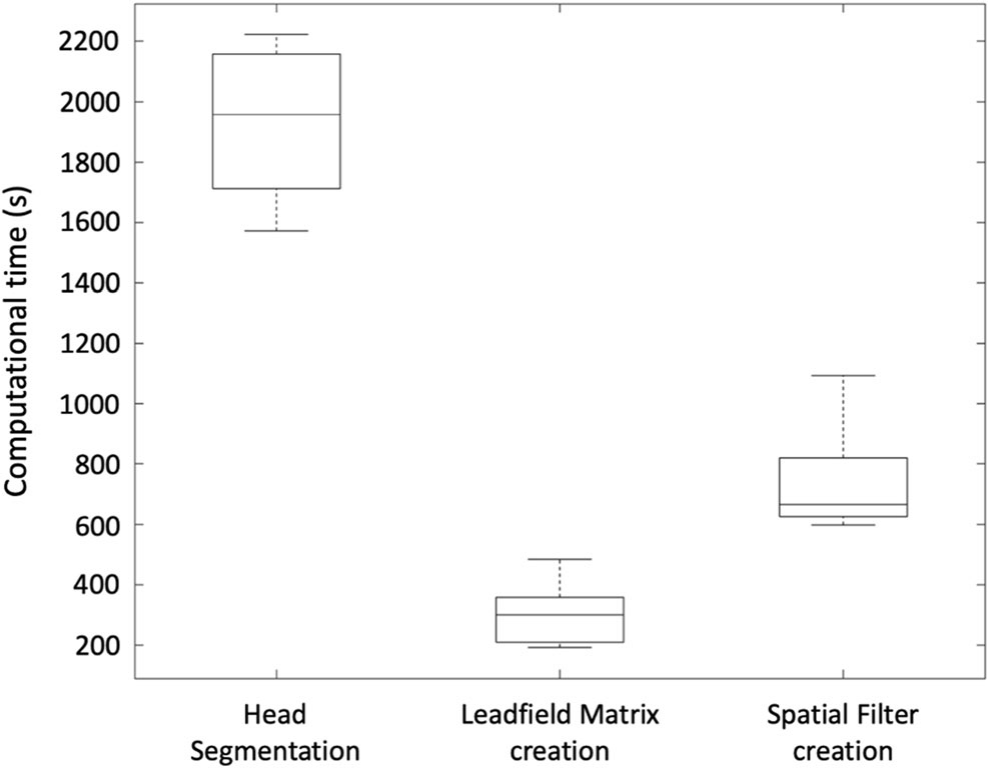
Computational time of RT-NET for the *Head segmentation*, *Leadfield matrix creation* and *Spatial filter creation* modules. The boxplots show the processing time calculated for each module, across all participants.

Computational efficiency is a key feature of RT-NET, which was specifically designed to support real-time processing of hdEEG recordings, so we quantified processing times for the different analysis stages. Average processing times for *Head segmentation*, *Leadfield matrix creation* and *Spatial filter creation* were 1938 s, 302 s and 735 s, respectively (Fig. 3). The first of these three modules should be used before the real-time EEG acquisition can start. The time required for the second and third modules should be kept as short as possible. Notably, the processing times we obtained for each of these two modules permit their execution during the EEG experimental session, and before the actual experiment. Besides the time required for preparatory steps, it is also important to consider the computational efficiency for real-time acquisition and processing. During our data collection, we measured acquisition time and delay. We divided our 6-minutes recordings, collected at a sampling rate of 1 kHz, into windows of 500 ms. Across all of them, the maximum delay introduced by real-time processing for artifact attenuation and source localization was 4 ms for each data buffer.

### Accuracy of RT-NET Analysis

**Fig. 4.**
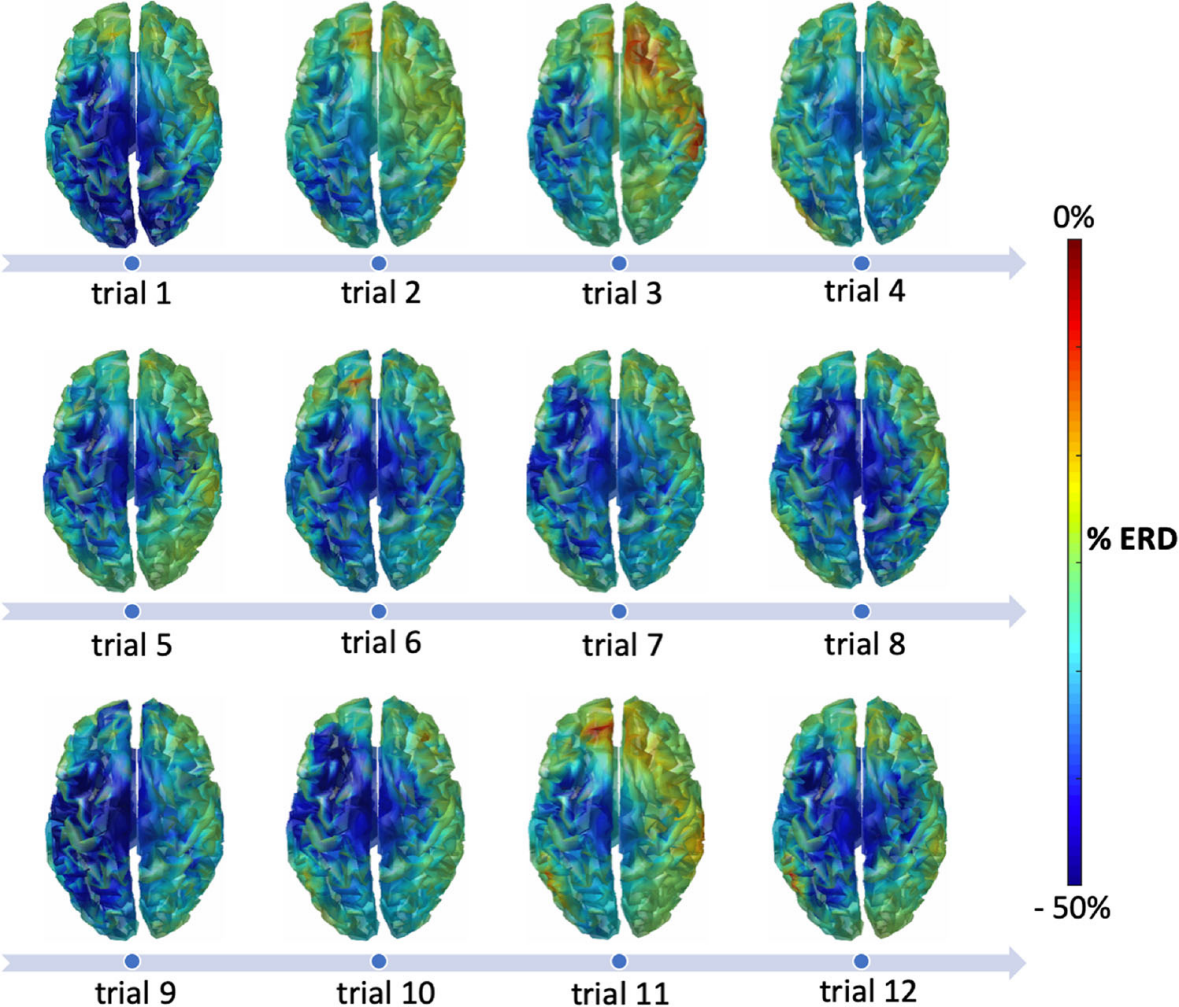
ERD maps in the beta band (13–30 Hz) for a single participant, obtained using RT-NET for 12 consecutive trials during right-hand movements. The maps are represented over a 3D cortical model in dorsal view.

**Fig. 5.**
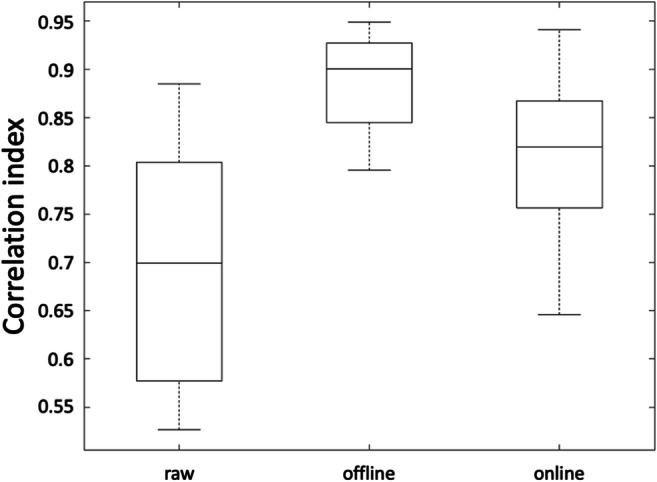
Spatial correlation of ERD maps in the beta band (13–30 Hz) calculated without artifact removal, offline and online processing, respectively. The boxplots show the average across-trial correlations across all participants.

**Fig. 6.**
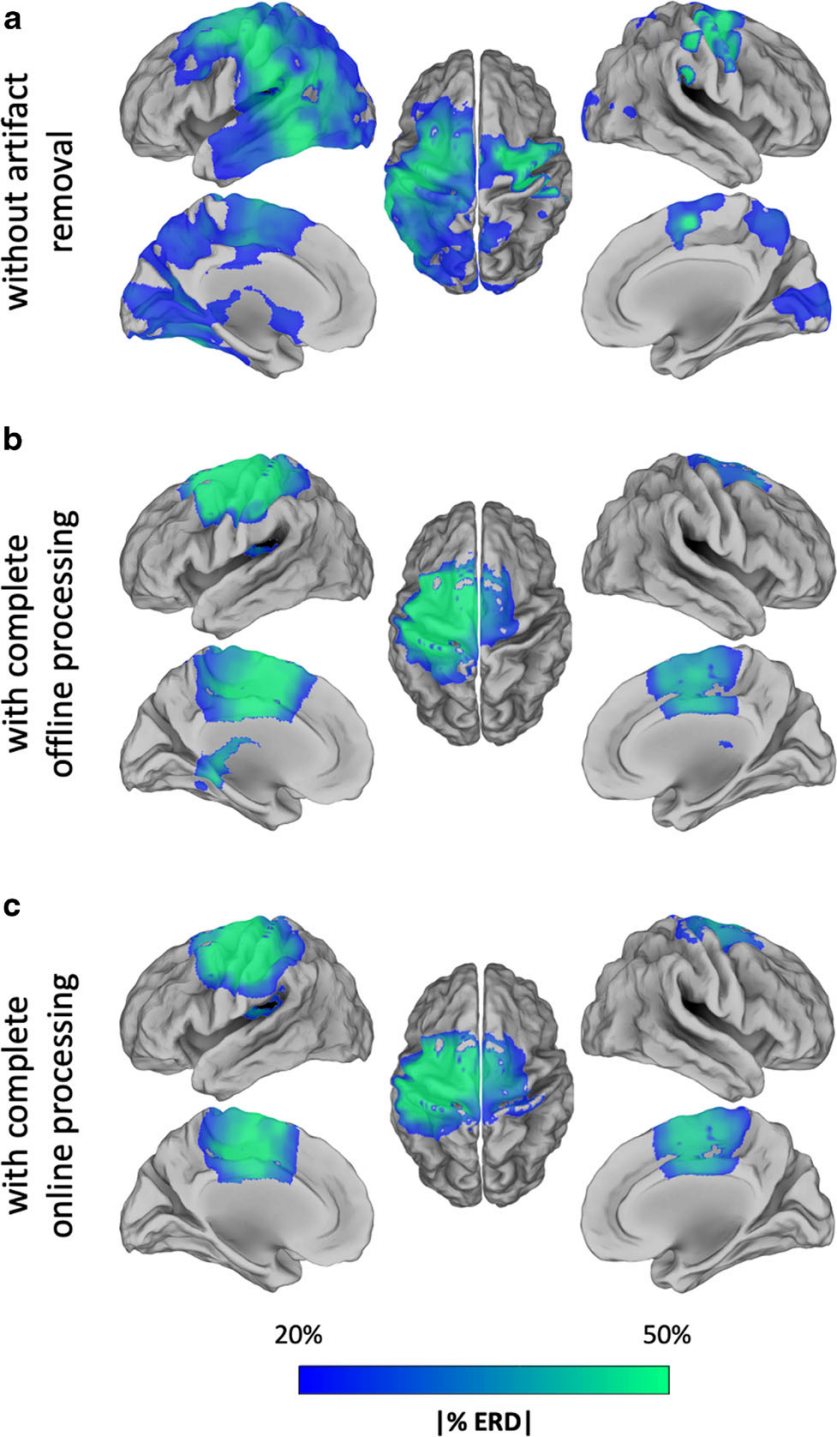
Motor-related power modulations in the beta band (13–30 Hz). (A) ERD map obtained without artifact removal (raw); (B) ERD map obtained with a complete offline processing workflow; (C) ERD map obtained by RT-NET. The maps are represented over a cortical surface in lateral, medial and dorsal views.

First, we examined the ERD maps produced online during the experiment for consecutive trials (Fig. 4). Each of them showed beta-band ERD peak within the primary motor cortex. The average spatial correlation of the ERD maps across trials was equal to 0.78. The correlation values obtained using EEG data processed with the online analysis workflow were not significantly different (Wilcoxon signed rank test, *p* = 0.06) from those obtained using ERD maps from an offline analysis (Fig. 5). Also, the beta-band ERD maps obtained using RT-NET were similar to those obtained using offline processing (Fig. 6). Quantitively, the correlation of group-level beta ERD maps obtained with RT-NET with the offline processing with and without artifact removal were equal to 0.76 and 0.56, respectively.

**Fig. 7.**
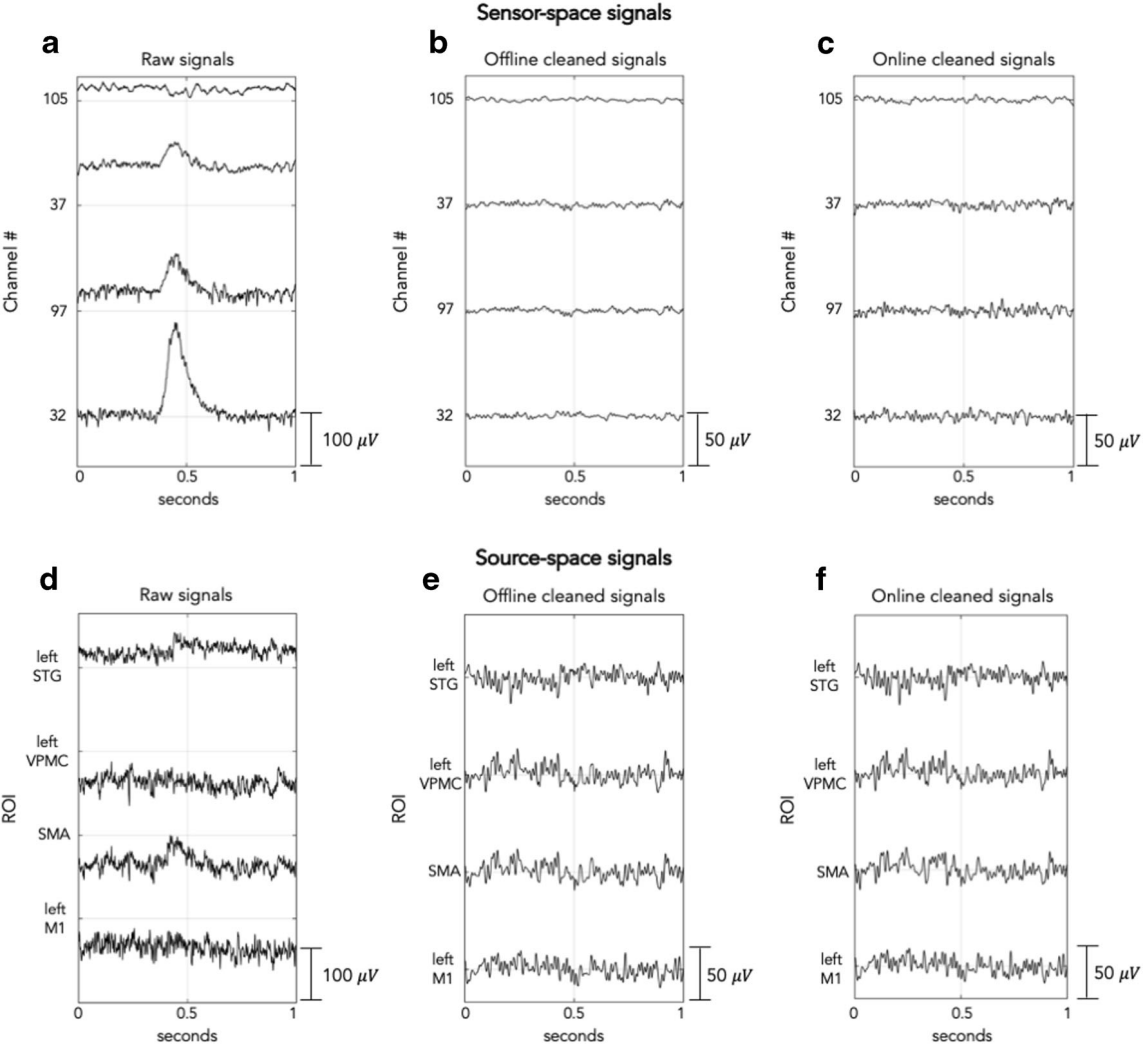
Representative examples of sensor-space (A–C) and source-space (D–F) signals from the same participant. Raw (A), offline cleaned (B) and online cleaned (C) EEG recordings in four representative channels; raw (D), offline cleaned (E) and online cleaned (F) voxel time-courses in left M1, SMA, left VPMC and left STG. M1: primary motor cortex; SMA: supplementary motor area; VPMC: ventral premotor cortex; STG: superior temporal gyrus.

**Fig. 8.**
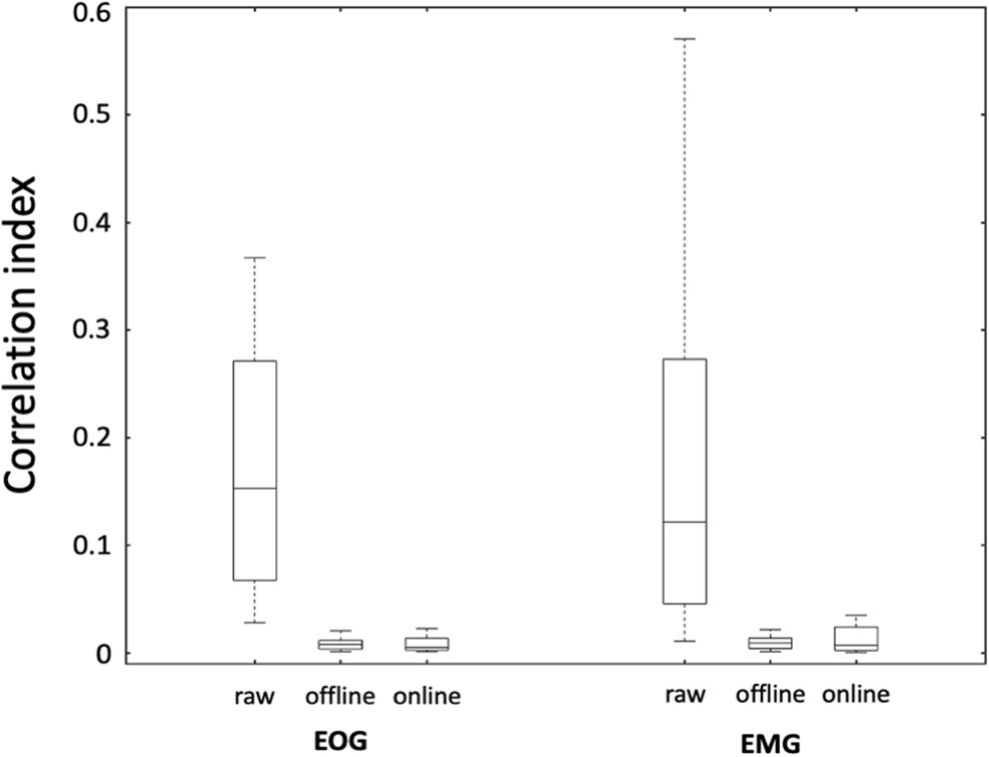
Absolute temporal correlation between EMG/EOG signals and neural time-courses. The analysis was conducted for neural signals reconstructed in left M1, SMA, left VPMC and left STG, without artifact removal, with online processing and offline processing, respectively. M1: primary motor cortex; SMA: supplementary motor area; VPMC: ventral premotor cortex; STG: superior temporal gyrus.

**Fig. 9.**
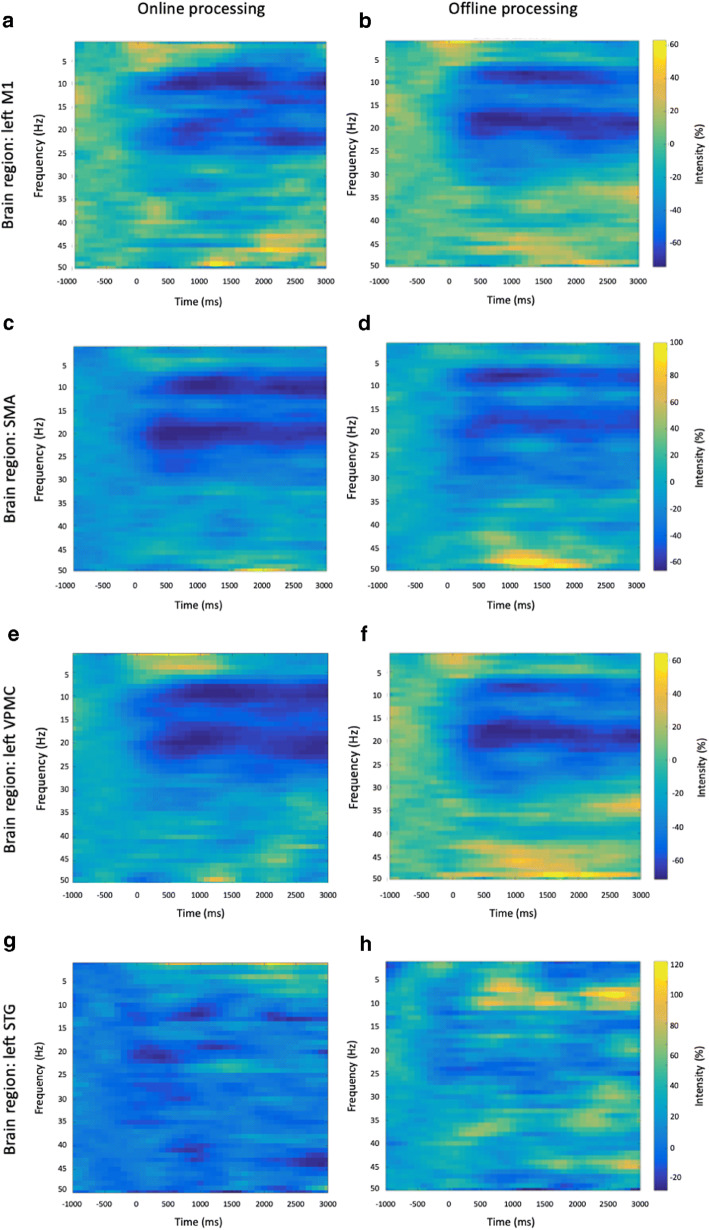
Single-subject ERD analysis for online (left column) and offline (right column) neural activity reconstructions. Power modulations are shown for left M1 (A and B), SMA (C and D), left VPMC (E and F) and left STG (G and H). In the left M1, SMA and left VPMC, there is considerable similarity between the online and offline methods in terms of desynchronization in the alpha and beta bands. As expected, the response in the left STG is hardly detectable. M1: primary motor cortex; SMA: supplementary motor area; VPMC: ventral premotor cortex; STG: superior temporal gyrus.

**Fig. 10.**
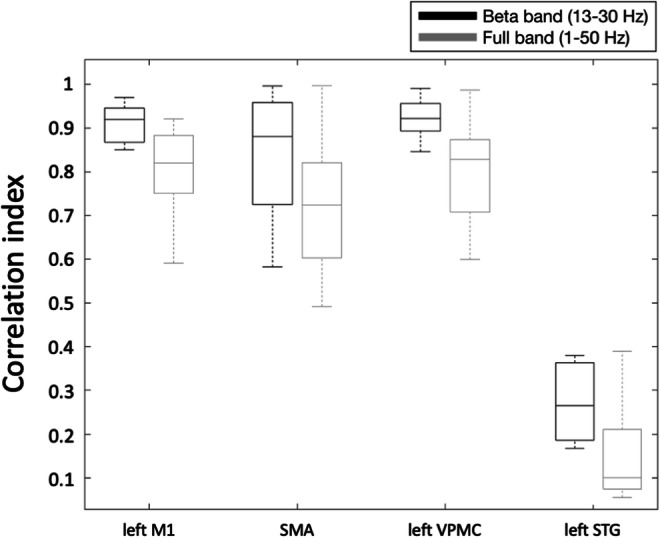
Correspondence of band-limited power time-courses between online and offline activity reconstructions in left M1, SMA, left VPMC and left STG. Comparisons were performed by examining the temporal correlation of band-limited power time-courses for the full band (1–50 Hz) and the beta band (13–30 Hz). A Wilcoxon signed rank test revealed higher temporal correlation for the beta compared to the full band for left M1 (***p*** = 0.0078), SMA (***p*** = 0.0156), left VPMC (p = 0.0078), but not for left STG (***p*** = 0.061). M1: primary motor cortex; SMA: supplementary motor area; VPMC: ventral premotor cortex; STG: superior temporal gyrus.

Similarities between online and offline processing were observed not only in the ERD maps, but also for time-courses reconstructed in three ROIs that are supposedly modulated by right-hand movements (left M1, SMA, left VPMC), and one that is likely not to be involved in task execution (left STG). Notably, there were no evident artifacts in the EEG data, after these were processed using the spatial filter of RT-NET (Fig. 7). The effectiveness of the online artifact removal procedure implemented in RT-NET was quantitatively assessed also by calculating the absolute correlation between reconstructed neural signals and EOG/EMG signals (Fig. 8). Values very close to zero were obtained for both online and offline processing, with no significant difference between them (Wilcoxon signed rank test, *p* = 0.5542 and *p* = 0.1923 for EOG and EMG, respectively). We then moved to the assessment of ERD after movement onset. Notably, a clear ERD could be detected in left M1, SMA and left VPMC, but not in the control region, left STG (Fig. 9). At the quantitative level, we observed that the correlation of power-modulations for the beta band (13–30 Hz), which primarily reflect motor-related neural activity, was significantly higher (Wilcoxon signed rank test, *p* < 0.05) than for the full band (1–50 Hz) in left M1, SMA and left VPMC (Fig. 10).

## Discussion

We have introduced RT-NET, a novel software package for real-time reconstruction of neural activity, which was specifically developed for the processing of hdEEG recordings. It includes a GUI that guides the user through the analysis steps and streams the processed data externally for real-time visualization or closed-loop applications. Below, we review the strengths and weaknesses of the software in comparison with alternative solutions and discuss the reliability of the results produced in our validation study.

### Primary Features of RT-NET and Comparison with Alternative Solutions

RT-NET relies on a single-window GUI (Fig. 2) that gives access to four different processing modules. They need to be run sequentially and are compatible with the different stages of a hdEEG experiment (Fig. 1). In previous studies, we have focused on methodological developments supporting the use of hdEEG as a brain imaging tool (Michel et al. 2004). In particular, we showed that combining high-density electrode montages with accurate head models enables more precise source localizations and thereby the reconstruction of brain network activity in the human brain (Liu et al. 2017, 2018). In the present study, we concentrated our efforts on developing novel solutions for the real-time reconstruction of brain activity using hdEEG. RT-NET relies on the online artifact removal method described in Guarnieri et al. (2018), which is initialized using a calibration dataset collected before the real experiment. This allows the creation of a spatial filter to be applied to the hdEEG data as they are acquired. This solution ensures a low computation time (Fig. 3), which makes RT-NET compatible with neural activity reconstruction.

RT-NET is not the only solution for acquisition and real-time source analysis from electrophysiological data. Indeed, MNE Scan and NeuroPype also provide comprehensive real-time analysis tools for EEG data, including preprocessing and source estimation. Notably, RT-NET has specific features that are not present in MNE Scan and Neuropype: it permits the creation of a realistic, individualized head model during the EEG experimental session, using the electrode positions and the T1-weighted MR image of the participant’s head. In particular, the MR image needs to be segmented to define individual head tissues. This processing step is accomplished in RT-NET using SPM12, which is also written in MATLAB. Another valid tool for MR segmentation is Freesurfer (https://surfer.nmr.mgh.harvard.edu) (Fischl 2012). Both FreeSurfer and SPM12 can provide volumetric measures from T1-weighted images, and a comparison between them has been performed in several studies (Fellhauer et al. 2015; Palumbo et al. 2019; Perdue and Diamond 2014). It has been reported that SPM12 is computationally more efficient than FreeSurfer (Henson et al. 2019; Schwarz et al. 2016), and provides more robust segmentations, except for the white matter (Guo et al. 2019).

Previous studies have already demonstrated that the use of individualised head models leads to better source localization results than templated head models (Akalin Acar and Makeig 2013; Brodbeck et al. 2011; Liu et al. 2018). Both MNE Scan and RT-NET calculate the forward model by means of boundary element method (BEM), which provides a realistically shaped volume conductor model without a significant increase in computational demand (Fuchs et al. 2002). Different BEM implementations are available, and in particular, RT-NET and NeuroPype rely on the symmetric BEM (sBEM) implemented in OpenMEEG (Gramfort et al. 2010, 2011). This solution outperforms other BEMs in terms of precision, but has relatively longer computation times (Adde et al. 2003; Clerc et al. 2010; Gramfort et al. 2011).

MNE Scan, NeuroPype and RT-NET implement different solutions for artifact attenuation, i.e. signal-space projection (SSP) (Uusitalo and Ilmoniemi 1997), Artifact Subspace Reconstruction (ASR) (Mullen et al. 2013) and a spatial filter based on ICA (Guarnieri et al. 2018), respectively. It has been shown that, in general, ICA-based artifact correction performs better than SSP (Haumann et al. 2016) and ASR (Kim and Kim 2018). Despite their low computational requirements, both ASR and our ICA-based approach require a calibration recording for reliable filter initialization. Notably, the combination of ASR and our ICA approach could certainly yield superior artifact removal performance than each method separately. However, since ASR and ICA would be to be applied sequentially, their computation times would sum up, and most likely become incompatible with real-time processing requirements.

The estimation of source activity by RT-NET is performed by eLORETA (Pascual-Marqui et al. 2011). However, RT-NET additionally includes MNE (Hämäläinen and Ilmoniemi 1994), sLORETA (Pascual-Marqui 2002), wMNE (Lin et al. 2006) and LCMV (Van Veen et al. 1997). In contrast, eLORETA, sLORETA and LCMV algorithms are implemented in NeuroPype, whereas MNE Scan can perform source localization with Real-Time Clustered Minimum-Norm Estimates (RTC-MNE) (Dinh et al. 2015) and Real-Time Clustered Multiple Signal Classification (RTC-MUSIC) (Dinh et al. 2017). There is no consensus about which EEG source localization algorithm is best to use, as this may largely depend on the signal-to-noise ratio of the EEG data, the EEG montage density and coverage, and the accuracy of the head model used (Michel et al. 2004). eLORETA has lower localization errors compared to LORETA and sLORETA (Jatoi et al. 2014), but has relatively low spatial resolution (Jatoi and Kamel 2017). Conversely, array signal processing-based algorithms such as MUSIC (Mosher and Leahy 1998) offer high resolution but at the cost of high computational complexity (Jatoi and Kamel 2017), with risk of data loss (Gaho et al. 2018). MNE (Hämäläinen and Ilmoniemi 1994) is less accurate than eLORETA (Im 2018), which is minimally affected by the volume conduction problem under real conditions (Pascual-Marqui et al. 2011). It may also fail in the localization of deep sources (Gaho et al. 2018).

RT-NET can stream data to other applications, as done in the current study (Fig. 4), such that the reconstructed neural activity can also be visualized in real-time. Generally speaking, RT-NET may be beneficial for novel BCI applications, such as source-based neurofeedback (Boe et al. 2014; van Lutterveld et al. 2017) and closed-loop neuromodulation techniques (Semprini et al. 2018).

### Validation of Real-Time Neural Activity Reconstruction by RT-NET

To validate the real-time reconstruction of neural activity using RT-NET, we employed hdEEG recordings collected during right-hand movements. Usually, simple motor tasks are expected to induce prominent contralateral modulation of brain activity. However, depending on task complexity, there may also be ipsilateral modulation. The specific motor task involved in our study is expected to elicit prominent contralateral modulation of brain activity, and in particular an event-related desynchronization in the beta band (Pfurtscheller and Lopes Da Silva 1999), in the hand representation of the left M1, in the SMA and in the left VPMC (Gorgolewski et al. 2013; Grodd et al. 2001; Lotze et al. 2000). The left STG, which was used as a control region, did not show substantial neural activity modulations. We performed an offline analysis of the hdEEG data using the approach we defined in previous studies (Liu et al. 2017). This served as a reference to assess the effectiveness of real-time neural activity reconstruction by RT-NET (Figs. 9 and 10).

The ERD map generated for the beta band showed the strongest values in the region covering the left M1, SMA and left VPMC for both online and offline processing (Fig. 6). The peak locations in the map were consistent with those previously reported in transcranial magnetic stimulation and fMRI studies (Gorgolewski et al. 2013; Hlustik 2001; Weiss et al. 2013). Furthermore, the neural signals reconstructed in the selected ROIs not only showed consistent task-related modulations, but also had very small artifactual contamination, as indicated by the absolute temporal correlation with EOG and EMG signals (Figs. 7 and 8).

Overall, the results of the present study suggest that the real-time processing of hdEEG data is sufficiently reliable, both in terms of spatial maps and in terms of the reconstructed time-course for specific brain regions. It should be noted, however, that we used hdEEG signals collected during a hand movement task, which is expected to produce robust neural responses. Further methodological work may be necessary to increase the sensitivity and accuracy of hdEEG-based studies in which brain sources produce less intense and more distributed signals. Our results support the idea that hdEEG can be used for solving brain dynamics with high spatial resolution. As such, hdEEG could be used as an alternative to fMRI for functional brain imaging, with the additional benefit of directly measuring brain activity. In addition, hdEEG can provide faster neurofeedback as compared to fMRI (Thibault et al. 2016).

### Limitations and Possible Caveats

A possible caveat of RT-NET may be the use of a relatively simple head modelling strategy, to keep the processing time compatible with that of a typical EEG experiment. Notably, whereas the MR image segmentation can be performed before the experiment, electrode positions need to be obtained from the participant before the leadfield matrix can be created. In this study we used a standard digitizing technique for extracting electrode positions, but it is worth noting that 3D scanning has been recently proposed to yield rapid and reliable electrode positioning (Taberna et al. 2019a; Taberna et al. 2019b). 3D scanning technology may be particularly useful for hdEEG systems, approximately halving the acquisition time. For the head modelling step, a three-layer sBEM model is currently used in RT-NET because of its relatively low computational demand. Whereas most BEM implementations rely on 3 layers (brain, skull, skin), the use of 4-layer BEMs that includes the cerebrospinal fluid (CSF) around the brain has been proposed to improve source localization (Akalin Acar and Makeig 2013). BEM solutions using 4 layers (Stenroos and Nummenmaa 2016) may be integrated in future versions of RT-NET, if their computation time will become as low as few minutes, such that they can be used in real-time EEG experiments. It should also be noted that head modelling approaches other than BEMs are also used by the neuroimaging community: finite element methods (FEMs) and finite difference methods (FDMs) (Hallez et al. 2007). FEMs and FDMs can take advantage of a more refined head segmentation than BEMs, and typically yield more precise estimates of the leadfield matrix. However, due to their computational requirements, they are not compatible with the creation of a head model in the course of an EEG experiment. Future methodological developments for parallelized FEM and/or FDM computations (Cuartas Morales et al. 2019) are warranted to reduce processing times and make them compatible with the requirements of real-time EEG experiments using RT-NET. Furthermore, RT-NET performs online artifact attenuation as well as source localization. To optimize artifact attenuation, we recently proposed a method relying on a calibration dataset (Guarnieri et al. 2018). This calibration dataset should contain a sufficient number of artifactual occurrences for an effective setup of the spatial filter. Although it remains difficult to determine how long the calibration dataset should be, it may be helpful to ask the participant to intentionally generate such artifacts (Zhang et al. 2015). Finally, we would like to point out that a more extensive validation of RT-NET using different tasks and experimental conditions, would be very important. In this study, we have tested RT-NET using hdEEG data obtained during motor task performance. Further work should extend the validation to hdEEG data during auditory stimulation, such that it would be possible to examine the performance of RT-NET when bilateral sources are active.

## Conclusions

RT-NET is a toolbox for the online reconstruction of neural activity from hdEEG signals. It has been specifically conceived and designed to support real-time analyses in the source space. This makes it unlike most software that, given the high computational demand of hdEEG processing, can only support offline source-space analyses. Notably, the accuracy of online neural activity reconstruction by RT-NET is comparable to that achieved with offline processing. We hope that our software package will contribute to the development of novel BCI applications based on hdEEG, such as source-based neurofeedback (Boe et al. 2014; van Lutterveld et al. 2017). Our future research endeavor will be directed towards an extensive validation of RT-NET in a wide range of real-time hdEEG experiments.

### Information Sharing Statement

RT-NET software is distributed according to a GNU General Public License, and is available for download at https://www.nitrc.org/projects/rtnet and https://github.com/robertoguarnieri/rtnet.
